# Targeting PR1 in myeloid leukemia

**DOI:** 10.18632/oncotarget.23403

**Published:** 2017-12-18

**Authors:** Gheath Alatrash, Jeffrey J. Molldrem, Muzaffar H. Qazilbash

**Affiliations:** Department of Stem Cell Transplantation and Cellular Therapy, Associate Professor of Medicine, Section of Transplant Immunology, Houston, TX, USA

**Keywords:** PR1, leukemia, immunotherapy, vaccine, AML

Acute myeloid leukemia (AML) remains an incurable malignancy. Although the incidence of AML is approximately 20,000 cases annually, the 5-year survival rate remains low at nearly 30%. There are a number of promising therapies for AML currently under development, especially in the relapsed and refractory settings. However, to date, there have been very few breakthroughs in the treatment of relapsed or refractory AML. Allogeneic hematopoietic stem cell transplant (allo-HSCT) remains a cornerstone therapy for patients with AML, especially patients with aggressive disease. However, allo-HSCT carries a high risk of mortality and morbidity due to the toxicities associated with conditioning regimens used prior to infusing the donor cells, and graft-*versus*-host-disease (GVHD), which can affect up to 50% of allo-HSCT recipients and is a major cause of post-allo- HSCT morbidity and mortality. Conversely, the incidence of GVHD directly correlates with the graft-*versus*-leukemia (GVL) effect and achieving disease remission. This observation is likely due to the donor T cells targeting antigens that are shared between the AML blasts/stem cells and normal tissue. Despite decades of laboratory and clinical research, there remains a paucity of antigens that are known to be associated with GVHD and/or GVL. Yet, separating GVHD from GVL is critical to the success of future targeted therapies for AML.

Our data, along with studies from a number of other investigators, have identified myeloid azurophil granules as a source for immunogenic antigens in AML [1–3]. PR1, a nonameric human leukocyte antigen (HLA)-A*0201- restricted peptide derived from the azurophil granule proteases neutrophil elastase (NE) and proteinase-3 (P3), has been the prototype peptide target in myeloid malignancies. Both NE and P3 have been shown to be highly expressed by myeloid malignancies, including AML, MDS and CML. A number of therapies have been developed that target PR1, including PR1-peptide vaccine [4, 5], anti-PR1/HLA-A2 antibody [6], and PR1-specific cytotoxic T lymphocytes (CTL)[7]. The latter two remain in preclinical development (Figure 1).

**Figure 1:**
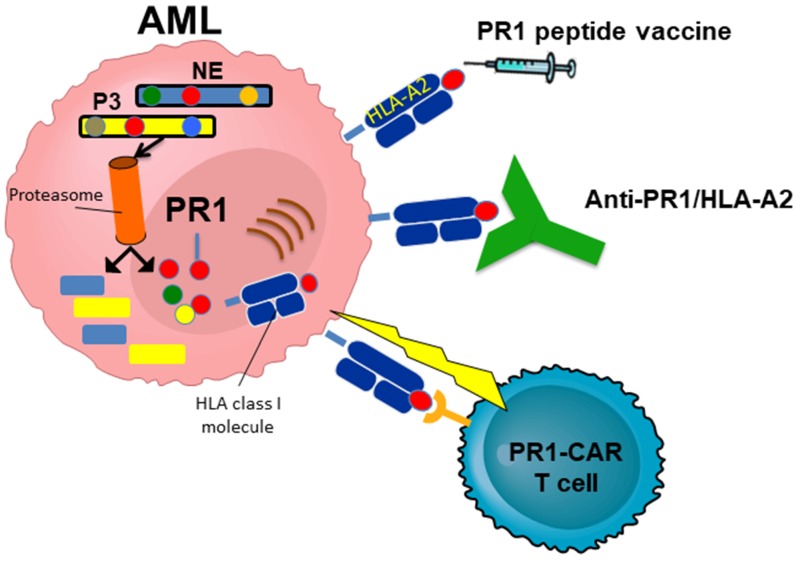
Therapies targeting PR1 PR1 is derived from neutrophil elastase (NE) and proteinase 3 (P3), and is presented on human leukocyte antigen (HLA)-A*0201. Although PR1 peptide vaccine is the only PR1-targeting therapy that has been tested in clinical trials, a number of therapies have been developed to target PR1 and have shown promise in pre-clinical *in vivo* models, including a T cell receptor (TCR)-like monoclonal antibody and a chimeric antigen receptor (CAR) T cell that target PR1/HLA-A2.

We reported results from a phase I/II dose escalation clinical trial investigating the toxicity, as well as clinical and immunologic responses following PR1-peptide vaccine in 66 patients with AML (*n* = 42), MDS (*n* = 11) or CML (*n* = 13) [4]. Our results showed no grade 3 or 4 toxicity in any of the patients vaccinated. The primary toxicities noted were grade 1 (*n* = 30) and grade 2 (*n* = 4) injection-site reactions. In addition, there was no worsening of GVHD in any of the 16 patients who had received allo-HSCT prior to vaccination, and none of the patients developed anti-P3 antibodies (cANCA), an important observation considering that PR1 peptide is derived from P3. An immunological response (defined as a ≥2-fold increase in the frequency of PR1-CTL) was seen in 53% of patients (*n* = 35), did not correlate with the PR1 peptide vaccine dose or the number of vaccinations received, and occurred soon after the receipt of the vaccination (by 3 weeks in 89% of the patients). Importantly, having < 5% bone marrow blasts (*P* = 0.01) and younger age (*P* = 0.03) correlated with achieving an immunologic response (IR).

These data are critical for vaccine therapy for AML, as well for PR1-targeting approaches. First, we showed that an immune response can be elicited in patients with AML. This is an important finding since the AML microenvironment has been reported to be immunosuppressive due to quantitative and qualitative factors. Nevertheless, the correlation between lower AML burden and achieving an IR highlights a window where vaccinations may be effectively administered to patients to elicit anti-AML immunity. Second, our data point to the effectiveness of targeting the leukemia-associated antigen, PR1. In our study [4], PR1-CTL were detected in 9 of 25 clinical responders *versus* 3 of 28 clinical non-responders (*P* = 0.03). Thirdly, the data highlight the safety of targeting PR1 in hematologic malignancies, an observation that is highly significant for the future of PR1- targeting approaches since the PR1 epitope is expressed by normal hematopoietic cells, although at a much lower level [8]. Based on these results, we can confidently conclude that targeting PR1 is a safe and effective strategy for the treatment of myeloid malignancies, particularly in patients with a minimal disease burden to consolidate remissions. The ideal time of vaccination, however, and the use of adjuvants following vaccinations could significantly improve the efficacy of vaccines.

## References

[1] Molldrem JJ (2000). Nat Med.

[2] Zhang M (2013). Clin Cancer Res.

[3] Kumari S (2014). Proc Natl Acad Sci U S A.

[4] Qazilbash MH (2017). Leukemia.

[5] Rezvani K (2008). Blood.

[6] Sergeeva A (2016). Leukemia.

[7] Ma Q (2016). Cytotherapy.

[8] Sergeeva A (2011). Blood.

